# Acid-Free Hydrothermal-Extraction and Molecular Structure of Carbon Quantum Dots Derived from Empty Fruit Bunch Biochar

**DOI:** 10.3390/ma13153356

**Published:** 2020-07-29

**Authors:** Norhanisah Jamaludin, Tong Ling Tan, Alif Syafiq Kamarol Zaman, Amir Reza Sadrolhosseini, Suraya Abdul Rashid

**Affiliations:** 1 Materials Processing and Technology Laboratory, Institute of Advanced Technology, Universiti Putra Malaysia, Serdang 43400, Selangor, Malaysia; anishanisah.jamaludin@gmail.com (N.J.); alifsyafiqkz@gmail.com (A.S.K.Z.); 2 Functional Device Laboratory, Institute of Advanced Technology, Universiti Putra Malaysia, Serdang 43400, Selangor, Malaysia; amir.reza@upm.edu.my; 3 Department of Chemical & Environmental Engineering, Faculty of Engineering, Universiti Putra Malaysia, Serdang 43400, Selangor, Malaysia

**Keywords:** carbon quantum dots, empty fruit bunch, hydrothermal, extraction, photoluminescent, DFT

## Abstract

Carbon quantum dots (CQD) have great potential to be used in various applications due to their unique electrical and optical properties. Herein, a facile, green and eco-friendly hydrothermal method for the preparation of carbon quantum dots was achieved using empty fruit bunch (EFB) biochar as a renewable and abundant carbon source. In the current study, the role of the hydrothermal process was observed and studied by comparing the morphology and optical characteristics of CQD obtained from EFB biochar. Interestingly, based on the high-resolution transmission electron microscopy (HRTEM) result, a considerably similar carbon quantum dots structure can be observed for the EFB biochar sample, showing the similar size and distribution of CQD. To further discuss the extraction of CQD from EFB biochar, a mechanism based on hydrothermal-induced extraction of CQD is proposed. The optimal structure of CQD deduced by density functional theory (DFT) in energy and dipole momentum was about 2057.4905 Hatree and 18.1699 Debye, respectively. This study presents a practical experimental approach in elucidating the molecular structure of photoluminescence CQD based on the Fourier transform infrared (FTIR) spectroscopy, X-ray photoelectron spectroscopy (XPS) and transmission electron microscopy (TEM) results.

## 1. Introduction

Carbon quantum dots (CQD) are a highly attractive class in carbon-based nanomaterials families which include fullerenes, nanotubes, nanodiamonds and graphene. CQD were first reported in 2004 by Xu et al. [1] via separation of single-walled carbon nanotubes through electrophoresis. Generally, CQD are zero-dimensional (0D) carbon nanoparticles with a sizes less than 10 nm, which exhibit similar properties to those of semiconductor nanoparticles [2]. In the ever-expanding nanomaterials research, CQD have received tremendous interest among researchers due to their fascinating properties such as highly biocompatibility, low-toxicity, ease of production, good solubility, chemical inertness, inexpensiveness and excellent photoluminescence [3,4]. Owing to these properties, CQD are widely used in multidisciplinary fields such as optoelectronics, photocatalysis, solar cells, bioimaging, drug delivery and photovoltaics [5].

The current worldwide research on CQD is particularly concentrated on two major themes; (1) application of newly discovered starting materials for CQD production; and (2) tuning synthesis to be more controllable and reproducible. Collective findings from these themes might be able to match the understanding of the structure of semiconductor quantum dots and finalise the mechanism of CQD formation and the governing principal of their photoluminescence (PL) properties [6]. Another key element that has placed CQD into the limelight is that production of CQD can be scalable and normally a one-step route using biomass waste-derived sources is involved [7].

Numerous methods have been proposed for the synthesis of CQD with outstanding optical and electronic properties. Two common approaches for the synthesis of CQD are high-energy ion beam radiation and laser radiation that use cement and graphite powders as starting materials [8,9]. Synthesis of CQD using a laser ablation technique is a fast-synthetic route however this method is economically not preferable and complicated operation. Chemical methods have been widely used for preventing the used of expensive precursors and energetic systems. Also, oxidation of gas soot, carbon soot or activated carbon with strong acids such as nitric acid are relatively inexpensive ways to synthesize CQD [10,11]. However, the usage of vast quantities of hazardous strong acids in the process is not favored. Meanwhile, the carbonization of molecular precursor materials such as glucose, sucrose, glycol, glycerol, citric acid, ascorbic acid to produce fluorescent CQD has attracted significant attention among researchers. Nonetheless, multiple-step processes and acids as well as post-treatments with surface passivating agents are required during the carbonization methods [12,13]. Currently, there are studies reported that one step hydrothermal carbonization with high temperature or microwave assisted hydrothermal carbonization using different carbon precursors can achieve self-passivating CQD [14,15,16,17,18]. Unfortunately, the major drawbacks of these methods are harsh synthesis conditions, complicated process, and the fact that they are highly expensive and time-consuming [19,20,21,22,23,24]. Despite these disadvantages, hydrothermal process is still preferred by researchers in preparing CQD as the process can be modified in order to make it facile and green.

Nowadays, a green synthesis approach has paved the way towards sustainable synthesis of CQD by focusing on the design of products and processes that minimise the use of harsh strong chemicals in the reaction. The production of CQD from renewable cheap natural precursors is a challenging but worthy concept. The usage of renewable starting materials and environmentally friendly non-toxic chemicals is one of the primary objectives of this approach. The reported work on CQD show a trend where there is a linking in the sequence of: carbon source–synthesis route–CQD structure–PL properties. The sources of carbon can be varied, inexpensive and abundant [25]. Table 1 summarises several synthesis methods of CQD using modified hydrothermal routes under different conditions. The methods listed use biomass and food waste as the carbon source and results in relatively small CQD which lie in the range of 1.5–10 nm.

In this study we report a facile and acid-free hydrothermal process to extract CQD from empty fruit bunch (EFB) biochar as the natural source of carbon. EFB biochar has traditionally been used as an effective adsorbent as well as a soil conditioner for agriculture. This process is scalable and the use of EFB biochar is convenient due to its abundance and ease of availability. It is important to note that Malaysia is the second largest palm oil producers after Indonesia and has an abundance of oil palm residues (~135 million tonnes) generated throughout the year. The palm oil wastes include EFB, palm oil fonds, mesocarp fiber, palm oil trunks and palm kernel shell. Among these, EFB is the highest wastes being produced (~69.87 million ton) worldwide in year 2017 [26]. The high volume of oil palm biomass residues incurs high disposal costs. Therefore, the utilization of EFB biomass that is abundantly available becomes the main focus of our study.

In a previous work, the hydrothermal process using salt bath of producing CQD from various types of biochar such as coconut shell, kenaf and EFB biochar had been carried out. It was found that CQD prepared from hydrothermally EFB biochar at subcritical water temperature of 250 °C yielded the highest fluorescence intensity, brightest blue fluorescence colour and smallest nanoparticle size [27]. The hydrothermal extraction of CQD from biochar involves no harsh chemicals or complex purification process. Hydrothermal treatments can generate a combination of pressure and temperature effect in the EFB precursors, leading to the uniform formation of separate CQD. Due to the interaction of CQD with polar solvents, a series of emission traps are dominated, and more functional groups are attached on CQD resulting in the highest luminescent intensity of CQD. However, the hydrothermal synthesis mechanism of CQD was not studied in detail. We propose that CQD can be extracted from the carbon source by the synergistic effects of the applied thermal process and intercalation of the co-solvent into the graphitic structure of the carbon source. Density functional theory (DFT) studies were also included in this work for the better understanding in terms of the most stable and optimal molecular structure formation of CQD, which we believe has not been previously reported.

## 2. Materials and Methods

### 2.1. Materials

EFB biochar was obtained from Pakar Go Green Sdn Bhd. Isopropanol (IPA, 99% assay) was purchased from Merck (Darmstadt, Germany). All chemicals were of analytical grade and were used as received without any further purification. Deionised water was used throughout the experiment.

### 2.2. Experimental

**Figure 1:**
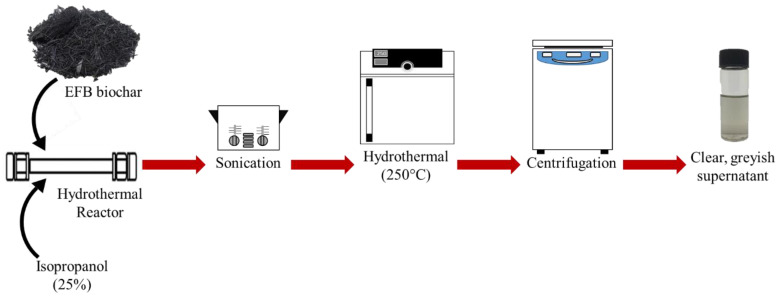
Schematic illustration of acid-free hydrothermal process from empty fruit bunch (EFB) biochar.

Biochar obtained was ground into small, fine powder using a pestle and mortar. A typical procedure for the hydrothermal-extraction of CQD is as follows. A known amount of EFB biochar was placed into a 10 mL steel tube followed by water and isopropanol (IPA) co-solvent in a 75%:25% ratio. The tube was tightly closed using Swagelok caps and sonicated for 5 min. Then, it was placed in the oven at a temperature of 250 °C for 1 h and the tube was then allowed to cool down by immersing it in a water bath. A black solution in the steel tube was centrifuged at 6000 rpm for 10 min to remove larger particles. A clear light greyish supernatant referred to as CQD suspension (CQD) was withdrawn for further characterization. CQD was further dialyzed using a dialysis tube (molecular weight cut-off (MWCO) of 3.5 kDa) against deionised water for three days, obtaining CQD solution (inside the dialysis bad) and dialysate (outside the dialysis bag). Dialysis is a widely used purification method to efficiently separate the CQD from the starting material and small fluorescent molecular by products for avoiding any erroneous conclusion in CQD research [28,29,30]. Studies indicate that CQD by-products will be trapped by the dialysis membrane with MWCO < 1 kDa, and hence MWCO = 0.5–1.0 kDa is not suitable for CQD purification [31]. The estimation concentration for the obtained CQD was 3000 ppm based on the excess biochar subtracted from the initial weight of EFB biochar. The attractive feature of this method is that neither harsh chemicals nor complicated process is involved. In order to demonstrate the importance of the hydrothermal process, the experiment was repeated without the hydrothermal step, but all other steps were kept the same. The resulting suspension obtained after centrifugation is referred to as EFB suspension (EFBs). The yield of CQD was calculated from the excess biochar subtracted from the initial amount of biochar. The typical conversion yield of CQD obtained was around 30% which is comparable to other top-down methods [32,33,34]. Figure 1 shows the schematic illustration of CQD preparation from EFB biochar using the hydrothermal route.

### 2.3. Material Characterization Methods

EFB biochar and CQD were characterised using attenuated total reflectance Fourier transform infrared (ATR-FTIR) spectroscopy at wavelength 400–4000 cm^−1^ (Thermo Nicolet 6700, Thermo Fisher Scientific, Waltham, MA, USA). The chemical bonding and binding energies were further determined using X-ray photoelectron spectroscopy (XPS) measurements with a PHI Quantera II with Spherical Capacitor Analyzer (SCA) and a monochromatic Al Ka (1486.6 eV) source. The morphology and particle size of CQD were investigated by high-resolution transmission electron microscopy (HRTEM) (FEI Tecnai G2 F20, Thermo Fisher Scientific, USA) operating at a higher accelerating voltage up to 200 kV. The sample preparation for HRTEM analysis was carried out with a small amount of carbon dots suspension dispersed in isopropyl propanol solution and sonicated for 5 min. Then, a drop of the suspension solution was placed on Cu grids covered with amorphous carbon film. Photoluminescence (PL) analysis (Perkin Elmer LS 55) was conducted to study the photoluminescence of CQD using Ar + laser with with Xenon discharge lamp at excitation wavelength of 300–425 nm. The absorbance of CQD was measured using an ultraviolet–visible (UV-Vis) spectrophotometer (Lambda 35, Perkin Elmer, Buckinghamshire, UK). The fluorescent lifetime of the CQD was measured using a time-resolved photoluminescence (TRPL) spectrum (HORIBA Instruments Incorporated, Edison, NJ, USA). The fluorescence transients were fitted with triple-exponential function: τ = A1 exp (−t/τ1) + A2 exp (−t/τ2) + A3 exp (−t/τ3), where A1, A2, and A3 are amplitude constituents of the first, second and third decay exponents.

### 2.4. Measurement and Calculation of Fluorescence Quantum Yield

The quantum yield of CQD was determined using a microplate reader (Infinite 200 PRO, Tecan, Männedorf, Switzerland). The quantum yield (φ) of CQD was determined by comparing the integrated PL intensities and the absorbance peak values of the CQD with quinine sulfate (QS) as a reference. QS was dissolved in 0.1 M H_2_SO_4_ and the CQD was dissolved in deionised water. The quantum yield of QS reported as 54% was selected as the standard reference due to the similar excitation and emission wavelength with the prepared CQD. The prepared CQD and QS were diluted to five different concentrations for absorbance measurement. In order to minimise re-absorption effects, absorption spectra of all samples under 0.1 at 300 nm was determined. In addition, their luminescent spectra with an excitation wavelength of 300 nm were also measured. The quantum yield was calculated using the following Equation (1) [35,36,37]:

*Q* = *Q*_st_(*I*/*I*_st_) (*n*/*n*_st_) (1)

where *Q*, *n*, *I* are the quantum yield, refractive index of solvent and integrated intensity of fluorescence spectrum, respectively. The subscript *st* refers to the standard sample.

### 2.5. Molecular Modelling Method

Molecular modelling of CQD was simulated based on the Fourier transform infrared (FTIR), HRTEM and XPS results to obtain the functional groups. Based on these results, the suggested 2D structure was drawn using ChemDraw software before its geometry was optimized. The ground state of CQD was calculated by using DFT with the Becke three-parameter Lee–Yang–Parr hybrid functional (B3LYP), CAM-B3LYP (long range) and 6-31G (p) basis set (B3LYP/6-31G(p)), CAM-B3LYP/6-31G(p) in singlet spin form [38,39,40]. In accordance with literature [41,42,43], the B3LYP is a suitable method for optimizing the molecule structure and finding the minimum energy. Gaussian 09 [44] was applied in DFT calculation by reducing the total energy without symmetry constraint. The 6-31G(p) basis set was found to be adequate in size by comparing geometric optimization and electronic properties with bigger basis sets [45,46,47]. Visualization of molecular orbitals and electron densities was undertaken with GaussView, version 5 [48]. The effect of solvent was considered during the calculation using solvation section and the Ionic Polymer Metal Composites I-PMC method was used. The mixture of solution (water and isopropanol) was defined using the “Self-Consistent Reaction Field SCRF” command. The presented calculations were based on the solvent effect.

## 3. Results and Discussion

### 3.1. Optical Properties of Carbon Quantum Dots (CQD)

**Figure 2:**
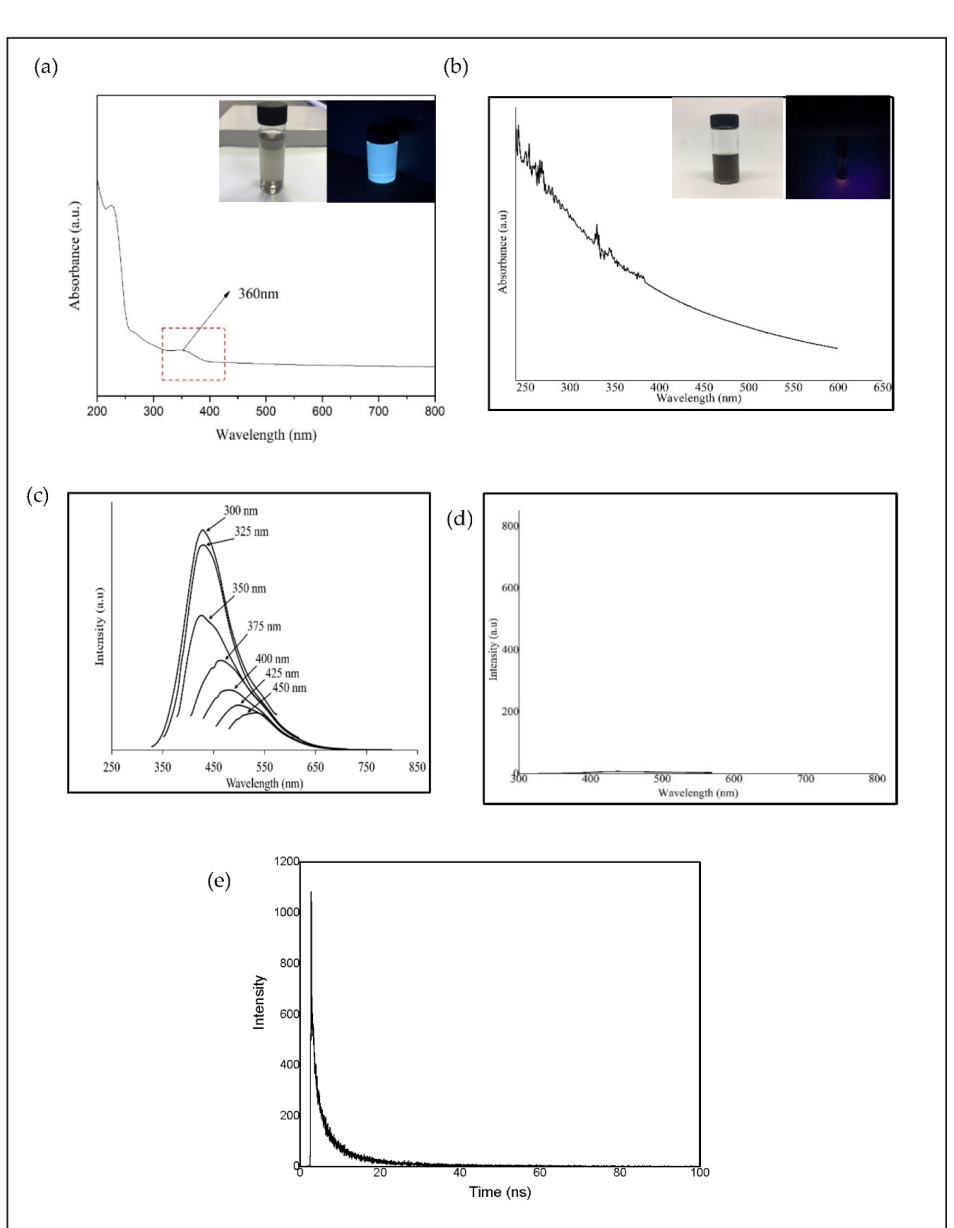
Optical spectra of supernatant obtained from (**a**) CQD and (**b**) EFB biochar (inset: images of supernatant under daylight and 365 nm ultraviolet (UV) light source); PL spectrum of (**c**) CQD at different excitation wavelengths (300–450 nm), (**d**) EFB biochar at excitation 300 nm and (**e**) fluorescence life time of CQD.

The optical properties of CQD were studied by using UV-Vis absorption and Photoluminescence (PL) spectroscopy and compared with the results of EFB biochar. The optical absorption peak of the CQD as shown in Figure 2a was observed in the UV region with an absorption peak at 226 nm and maximum absorption at 360 nm which is due to π-π* transition of the conjugated C=C bond and *n*-π* transition of the C=O band and [49,50,51]. It can be visualized that the CQD shows a different color in solution under daylight (greyish) and blue-green luminescence under the exposure of UV light (365 nm). Figure 2b shows the absorption spectra of EFB biochar. It can be seen that the EFB biochar suspension did not display any optical absorption peak and no color was observed under the exposure of UV light (365 nm) as shown in the inset of Figure 2b.

To explore the optical properties of CQD, a detailed PL study was carried out. The classic feature of CQD is the emission wavelength and size dependent photoluminescent behavior [19]. However, due to the complicated exact mechanism of the PL behavior of CQD, it remains an open debate. The feasible basics for the size-dependent PL behavior are due to the different particle sizes of CQD and the distribution of the different surface energy traps of CQD [18]. The difference in the position of emission peak is probably due to the variation in size of the CQD. With decreasing size of CQD, the energy gap will increase and vice versa due to the quantum confinement effect just like the traditional semiconductor quantum dots. The larger particle will be excited at relatively longer wavelengths while the smaller particle will be excited at lower wavelengths. Therefore, the intensity of the PL is highly relying on the number of particles excited at a particular wavelength [19].

The PL spectra of CQD as a function of excitation wavelengths (300–450 nm) are shown in Figure 2c. A strong PL emission peak located at 425 nm was observed with an excitation wavelength of 300 nm, most probably due to the largest number of particles which are being excited at this wavelength. The emission peak observed shows a shift to a higher wavelength (red shift) with the increase of the excitation wavelength. This indicates the dependency of emission and PL intensity to excitation wavelength. Other reason contributes for the excitation dependent PL behavior of CQD is the nature of their surface. The presence of functional groups such as hydroxyl, carbonyl and carboxyl on CQD might lead to a series of emissive traps between π and π* of C-C. Thus, the PL mechanism is speculated to be determined by both the size effects and the surface defects [19]. The PL spectra of EFB biochar at an excitation wavelength of 300 nm is shown in Figure 2d. It shows that the suspension obtained exhibits no peaks compared to the hydrothermally processed CQD. Without the hydrothermal process, EFB biochar does not fluoresce under UV light source.

The fluorescence lifetime curve of CQD is shown in Figure 2e. The lifetime data of CQD were fitted with a triple-exponential function as follows:

τ = A_1_ exp (−t/τ_1_) + A_2_ exp (−t/τ_2_) + A_3_ exp (−t/τ_3_)

where A_1_, A_2_, and A_3_ are amplitude constituent of the first, second and third decay exponents. The decay curve consisted of a rapid constituent (τ_1_) and two slow constituents (τ_2_ and τ_3_). The average fluorescence lifetime of CQD was 5.31 ns (τ_1_ = 0.0586 ns, τ_2_ = 1.8466 ns, τ_3_ = 9.0676 ns) in this study, which are higher than those of CQD prepared by pyrolysis and hydrothermal methods. The fluorescence lifetimes obtained were 3.18 and 2.78 ns as reported by Wang et al. [52] and Hong et al. [53]. According to Wang et al., the shorter lifetime of CQD obtained was caused by the defect states on the surfaces of CQD, suggests that these surface states could bring effects to the radiative recombination of electrons and holes and then energy is released in the form of photo emission. The nanosecond lifetime of CQD shows promise for use as nanoprobes in optoelectronic and biomedical applications.

**Figure 3:**
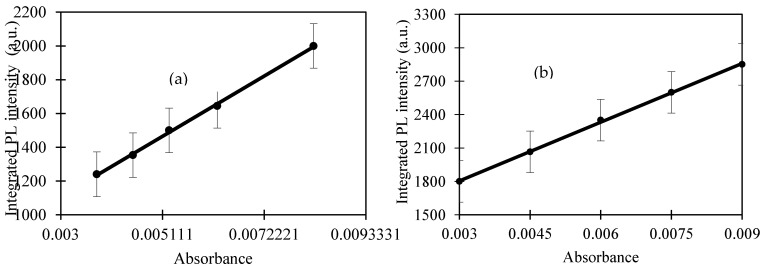
The variation of the integrated fluorescence with absorbance of (**a**) quinine sulfate (QS) and (**b**) CQD, respectively (number of replicates: 3).

The quantum yield is the ratio of number of photons emitted to number of photons absorbed by the sample. The comparative method [Equation (1)] is the reliable and famous method to obtain the fluorescence quantum yield. This method is based on comparative the wavelength integrated intensity of the test sample and a standard sample. The range of absorption of the standard sample should be matched with the range of the excitation wavelength of the test sample. As an increasing the accuracy of measurement, the variation of integrated intensity of fluorescence spectrum with absorbance at the excitation wavelength of the test sample and standard sample were used to calculate the quantum yield using Equation (1):

The gradient of solid lines was used to calculate the quantum yield using Equation (1) (as shown in Figure 3) and the refractive index was measured using surface plasmon resonance. Consequently, the refractive index of CQD and quinine sulfate were 1.371 and 1.4023, respectively. The quantum yield of CQD obtained in this study was 55.84% and we compared the quantum yield of CQD derived from other natural precursors, and their quantum yields are listed in Table 2. These results clearly reveal that the EFB biochar used in this study enhanced the quantum yields to a certain extent.

### 3.2. Spectroscopy Studies of CQD

#### 3.2.1. Fourier Transform Infrared (FTIR) Spectroscopy Analysis

**Figure 4:**
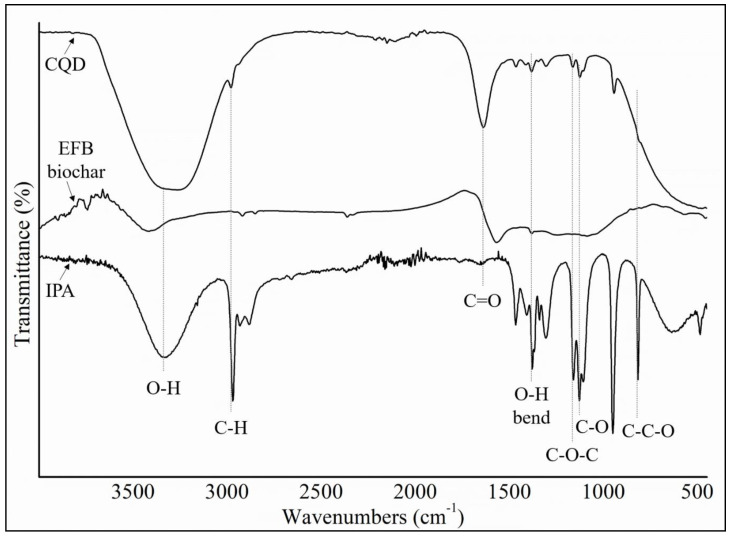
Comparison of isopropanol (IPA), EFB biochar and CQD Fourier transform infrared (FTIR) spectra.

FTIR spectroscopy was used to investigate the functional groups of CQD and EFB biochar together with IPA as a comparison, which are shown in Figure 4. FTIR spectrum of CQD showed a broad O–H peak observed at stretching vibrations of 3290 cm^−1^, C–H peak at 2970 cm^−1^, C=O peak at 1641 cm^−1^, C=C peak at 1421 cm^−1^, and C–O peak at 1047 cm^−1^. The FTIR spectrum showed the presence of oxygen-rich groups such as carboxyl and hydroxyl on the surface which explained the good water solubility of CQD [59]. In general, IPA has a broad and strong O–H stretch at 3350 cm^−1^ and the in-plane –OH bend at 1350 cm^−1^. For secondary alcohol, the C–O stretch bonding peak usually falls between 1150 cm^−1^ and 1075 cm^−1^. Thus, the peak at 1107 cm^−1^ can be assigned to the secondary alcohol of isopropanol and the peaks around 1163 cm^−1^ and 1107 cm^−1^ are assigned to the asymmetric stretching vibrations of C–O–C groups. The C–C–O symmetric stretch of isopropyl alcohol is at 817 cm^−1^ [60]. This versatility of surface functional groups opens up intricate pathways for the interaction between CQD and IPA solvent leading to the emission of bright blue fluorescence. It is known that surface oxidation creates surface defects, which play a role as capture centers for excitations and induces PL emissions [61]. After the hydrothermal treatment in the presence of IPA solvent, the FTIR spectrum of CQD shows stronger absorption bands of oxygen-related groups than EFB biochar. Meanwhile for EFB biochar, a broad peak at approximately 3400 cm^−1^, which is attributed to stretching vibrations of the O–H functional group was observed. Absorption peaks at 2921, 2851 and 1496 cm^−1^ were also identified which is attributed to the C–H stretching vibrations of the methyl group C–H band. Appearance of these C–H vibration peaks in the spectra of EFB biochar are attributed to the bending vibration of the C–H groups in the cellulose, hemicelluloses and lignin components, respectively.

#### 3.2.2. X-ray Photoelectron Spectroscopy (XPS) Studies of Empty Fruit Bunch (EFB) Biochar and CQD

**Figure 5:**
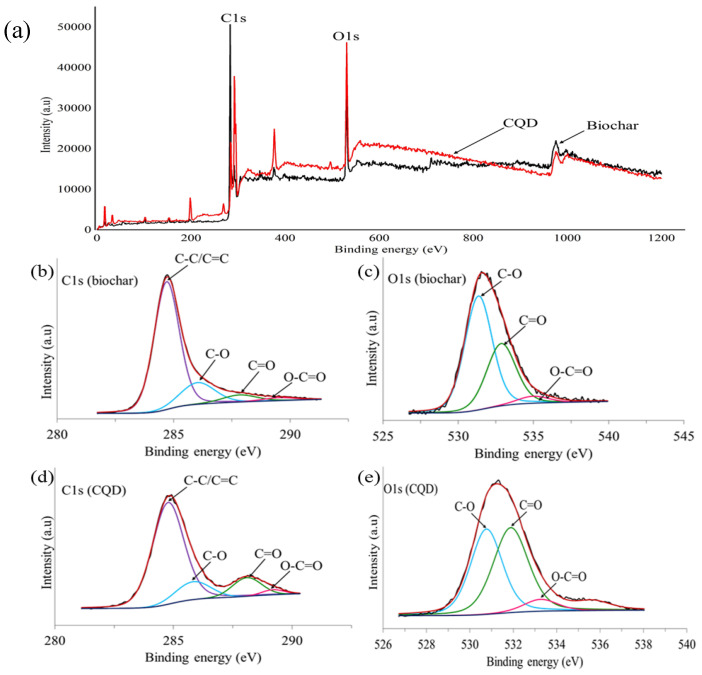
X-ray photoelectron spectroscopy (XPS) results related to CQD and EFB biochar, (**a**) full spectrum, (**b**) C1s spectra of EFB biochar, (**c**) O1s spectra of EFB biochar, (**d**) C1s spectra of CQD and (**e**) O1s spectra of CQD.

The CQD in this experiment was prepared from EFB biochar using hydrothermal treatment. Detailed component and chemical bonding studies about the EFB biochar and XPS are keys to determine the formation mechanism of CQD. Therefore, XPS analysis was performed to further investigate the existence of functional groups present on the surface of both EFB biochar and CQD. EFB biochar mainly consists of carbon and oxygen. The corresponding contents of these elements were 75.05% and 20.80%, respectively, as determined by XPS. The XPS results indicated that CQD are composed of atomic C (80.31%) and O (19.68%). As shown in Figure 5a, the XPS wide scan spectrum shows a dominant graphitic C1s peak and O1s peak of both EFB biochar and CQD at 292, 530 and 531 eV, respectively. The deconvoluted peaks of C1s in Figure 5b suggested the existence of four states of carbon elements in EFB biochar. The peaks consisted of C–C/C=C peak with a binding energy of 284.7 eV and attachment of oxygen functionalities such as C–O at 286 eV, C=O at 287.9 eV and O–C=O at 289.6 eV [62]. Similarly, the typical O1s spectrum in Figure 5c can be deconvoluted into three peaks at 531, 532.9 and 535 eV which are attributed to C–O, C=O and O–C=O respectively. From the C1s deconvoluted peaks of CQD in Figure 5d, four peaks are observed. The main peak at 284.8 eV is ascribed to C–C/C=C and the rest of the peaks are assigned to C–O, C=O and O–C=O groups. Figure 5e consists of three peaks, C–O, C=O and O–C=O as displayed in the O1s spectrum of EFB biochar. The XPS results clearly indicates that CQD were functionalized with hydroxyl, carbonyl and carboxyl groups [20] which brings advantageous to the surface modification and functionalization [63]. It is noted that the presence of carboxylate species due to the presence of oxygenic surface species are in good agreement with FTIR characterization results.

### 3.3. Morphological Properties of CQD

**Figure 6:**
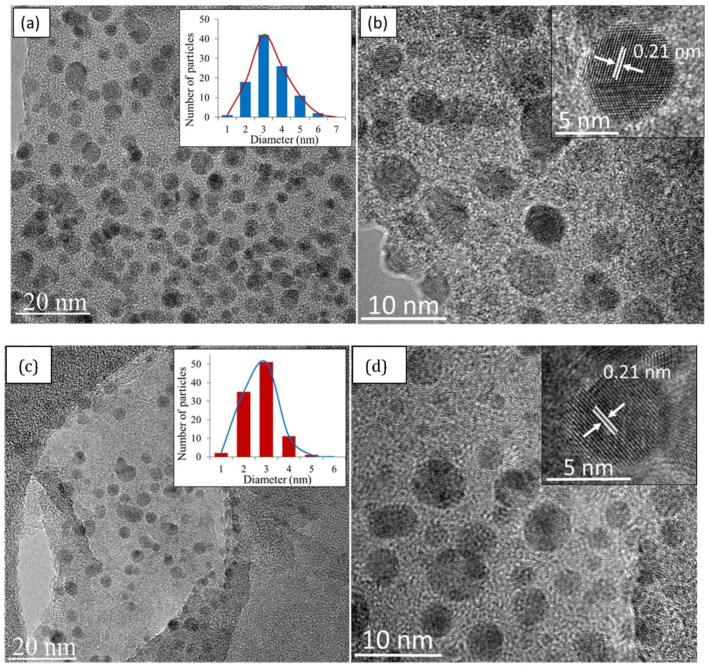
High-resolution transmission electron microscopy (HRTEM) images of (**a**) low magnification (inset: size distribution) and (**b**) high magnification of CQD (inset: lattice spacing); (**c**) low magnification (inset: size distribution) and (**d**) high magnification of EFB biochar (inset: lattice spacing).

HRTEM images of as prepared CQD are shown in Figure 6a,b. Figure 6a clearly shows that the size of CQD are small, nearly spherical in shape, and monodispersed. The histogram size distribution of CQD illustrated by the inset, shows that the majority of the dots lie in the range of 1–6 nm, with an average diameter of 3 nm (calculated from micrographs of 100 random particles). The high-magnification HRTEM of CQD depicted in Figure 6b, show that the dots exhibit good crystallinity with lattice fringes of 0.21 nm, which correspond to the (100) plane of graphitic carbon [50]. As shown in Figure 6a,b, the CQD obtained in this work were relatively uniform with a good crystalline structure.

In an attempt to understand the formation of CQD, it was imperative to view the EFB biochar starting material under HRTEM, which is shown in Figure 6c,d. Surprisingly, nanostructures very similar to CQD in terms of morphology and lattice spacing was observed. Initially, CQD was thought to be formed through an exfoliation mechanism of graphitic biochar nanoparticles into tiny carbon fragments. However, the presence of existing CQD in the starting material indicates that an extraction process is also at play. Note that CQD cannot be produced from pure EFB; only EFB biochar. This indicates that the process of pyrolysis to produce biochar also produces the carbonization of small nanostructures like CQD. The hydrothermal process did not change the physicochemical structure of the dots. To the best of our knowledge, there have been no previous reports of a hydrothermal route that uses organic solvent to directly extract fluorescent CQD from biochar.

### 3.4. Plausible Mechanism for the CQD Extraction

The purpose of this study was to observe the significance of the hydrothermal process in the facile one-step extraction of CQD from EFB biochar. Unlike other reported studies, this method applied a solid biochar from agricultural waste as a carbon source and a very common solvent as the liquid medium. The interesting feature of this approach is that neither harsh acids nor a post-synthetic surface passivation step is required. In our experiments, a temperature of 250 °C was chosen for the hydrothermal extraction of CQD. The basis of the hydrothermal reaction is the high solubility of substances in solvents at high pressure and temperature and the possibility of subsequent extraction of the dissolved material from the liquid phase. The relatively high temperature of water and IPA mixture compared to their respective boiling points, plays an essential role in the process. As the vapor pressure increases with temperature, the properties of the solvents in terms of diffusion rate and reactivity will change compared to that at room temperature. The surface and edges of CQD may consist of many oxygen-rich functional groups which were formed during the hydrothermal process enabling the extraction of CQD.

**Figure 7:**
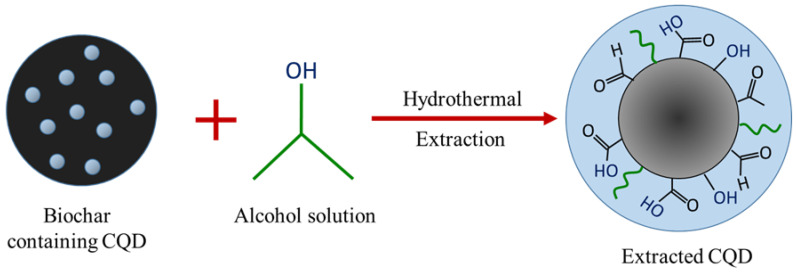
Possible mechanism for the extraction of CQD.

Figure 7 illustrates the formation mechanism of CQD via the one-step hydrothermal-extraction process. During the hydrothermal treatment, isopropanol bonds to the active surface of CQD, enabling CQD to be extracted out from the bulk biochar and dispersed in the liquid medium. The extracted CQD causes CQDs to fluoresce under UV light illumination as well as show high photoluminescence under different radiation wavelengths. The blue florescent CQD also reveal a broad PL peak that shifted along with the increase of excitation wavelength, which might due to the quantum-confinement effect and edge defects. The unique phenomenon of excitation dependent photoluminescence behaviour proved that the reaction products must be photoluminescence CQD, which was also in agreement with optical properties that were synthesised by other research groups [63,64,65]. In contrast, EFB biochar, which was obtained without the hydrothermal process, did not show any fluorescence, which indicates that CQD was essentially unable to be extracted from it. In addition, the role of the hydrothermal process can be well-explained based on the major difference of the peaks in FTIR spectra. EFB biochar is made up of carbonyl, carboxyl and hydroxyl groups as shown in the spectra. The same functional groups can be observed in the spectra of CQD after the hydrothermal process. As isopropanol acts as a dispersive medium, it can be seen that the peak of –OH bond shifted and the OH peak for the CQD structure was increased, indicating that there is an interaction between the functional groups of isopropanol and CQD surface groups. This interaction can also be confirmed through the shift of C1s and the increment of O1s peaks shown in XPS spectra. The similarity in composition and surface states of both biochar and CQD suggests that the hydrothermal process is able to extract out the quantum dots from the EFB biochar into the dispersive medium. As discussed earlier in the morphological study, it was shown that the EFB biochar itself already contains the dots in which the size and structure are virtually the same; almost spherical and monodispersed quantum dots with a range of 1–6 nm in size. The findings point towards the extraction of CQD during the hydrothermal process. Inspired by these aforementioned characterization results, CQD was successfully extracted with good water solubility and strong fluorescence properties in this work.

## 4. Molecular Modelling Based on Density Functional Theory

Many studies on the properties of CQD have been previously reported, however the limitation details of the molecular structure of CQD was due to the complexity and variety in shape and size of such dots. The molecular structure of the CQD is of special interest because there are many orientational possibilities of the functional groups moieties with respect to the aromatic ring. The concept of DFT Gaussian software is used to perform geometry optimization at ground state calculation of CQD for this study.

**Figure 8:**
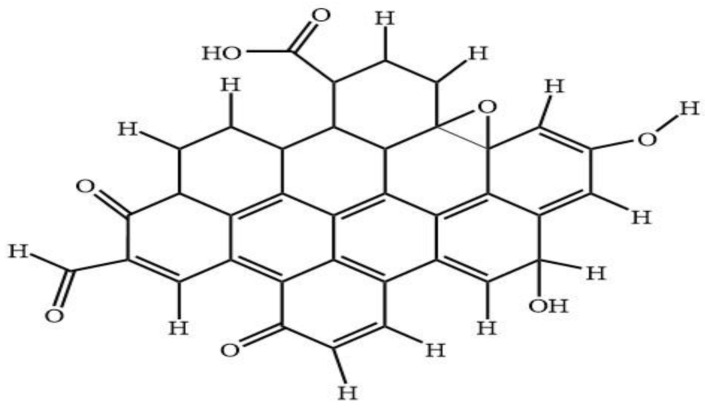
Proposed 2D structure of CQD.

Figure 8 shows the predicted model structure for DFT simulation of CQD. The CQD structure was constructed such that the terminal carbon atoms are attached by hydrogen atoms, hydroxyl and carboxyl group, to study the possibility of band edge position tuning. The method and basis set were DFT (B3LYP) [46,47,48,49] and 6-31G (p) in singlet spin form. The modelling was carried out in the ground state which means the lowest energy state. The simulation was undertaken vigorously from the maximum energy of the first structure until the minimum energy of the last structure was achieved. The simulation was done for different aromatic ring number including 9, 10 and 12 to find the best and optimum structure. The DFT calculation was dependent on charge distribution on the surface of molecular. In accordance with FTIR, XPS and TEM results, the molecular structure of CQD consists of C–O–C and C=C and the particle size was 3 nm.

The molecular structure was investigated using specific methods including Coulomb-attenuating method-3-parameter, Lee–Yang–Parr CAM-B3LYP and Becke, 3-parameter, Lee–Yang–Parr (B3LYP) with the base set as 6-31G(p). As a result, the molecule structure of CQD was derived from the simulation in the 103, 83, and 162 forms for 9, 10 and 12 rings, respectively. The parameters such as maximum force, root mean square RMS force, and RMS displacement were converged, and maximum displacement was not converged when the simulation was done using CAM-B3LYP method whereas all parameters were converged when the simulation was done under B3LYP method. The pertinent parameters are given in Table 3. The minimum energy, maximum energy, and dipole momentum for molecule structure of CQD with 9, 10, and 12 rings have been presented in the Table 4. Moreover, the FTIR spectra were derived from simulation of CQD with different ring number (9, 10 and 12).

**Figure 9:**
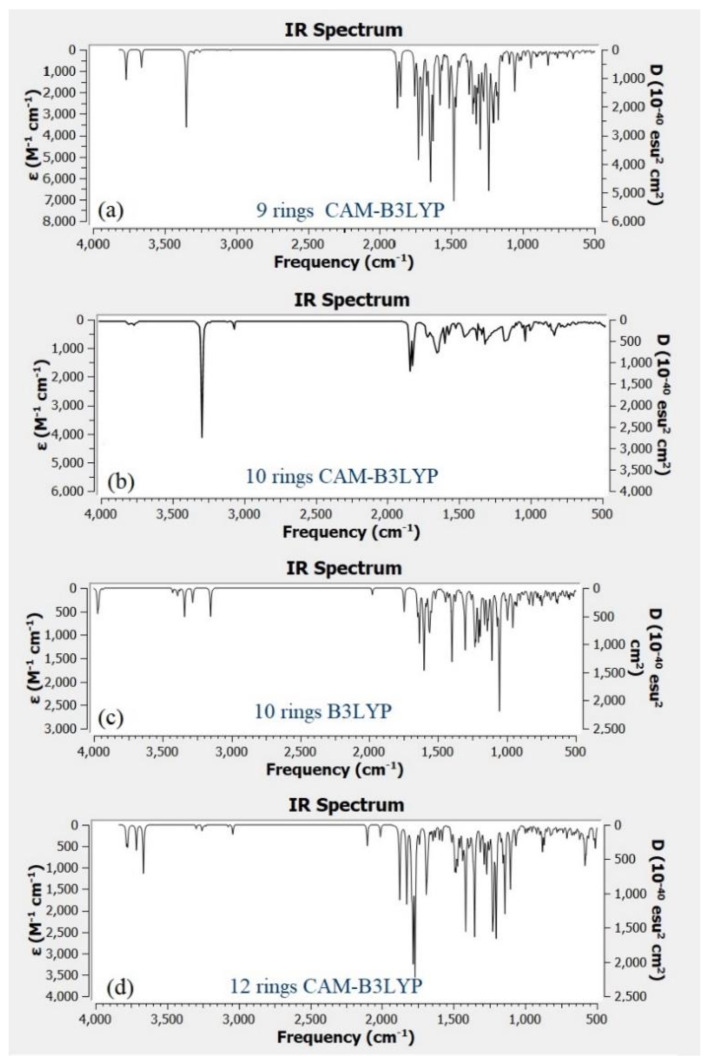
Infrared (IR) spectrum related to (**a**) CQD with 9 rings (CAM-B3LYP), (**b**) CQD with 10 rings (CAM-B3LYP), (**c**) CQD with 10 rings (B3LYP), (**d**) CQD with 12 rings (CAM-B3LYP).

Figure 9a–d shows the IR spectra for different structure of CQD. Based on the experimental FTIR result, the main peaks occurred at 3290, 1641, 1421, and 1047 cm^−1^. Figure 9b shows the main peaks of CQD at 3292, 2990, 1638, 1457, 1049 cm^−1^ assigned to the O–H, C–H, C=O, C=C, and C–O stretching vibrations, respectively. As a comparison with the experimental FTIR result as presented in Figure 4, the position and intensity peaks for CQD structure with 10 rings matched with the intensity and position of FTIR peaks in experimental results well. The FTIR simulation spectra for 9 and 12 rings show the main peaks in other position of the intensity peaks which are greater or lower than the experimental FTIR result for CQD. In the FTIR spectrum for 10 rings, not only the position of the peaks for O–H, C=O, C=C, and C–O stretching vibrations appeared near the experimental FTIR spectrum but also the peak intensity of FTIR spectrum are near to experimental results. Furthermore, the FTIR spectrum for CA-B3LYP method is better matched to experimental FTIR results that B3LYP method. As a result, molecule structure of CQD with 10 rings is a suitable model to propose structure of CQD.

**Figure 10:**
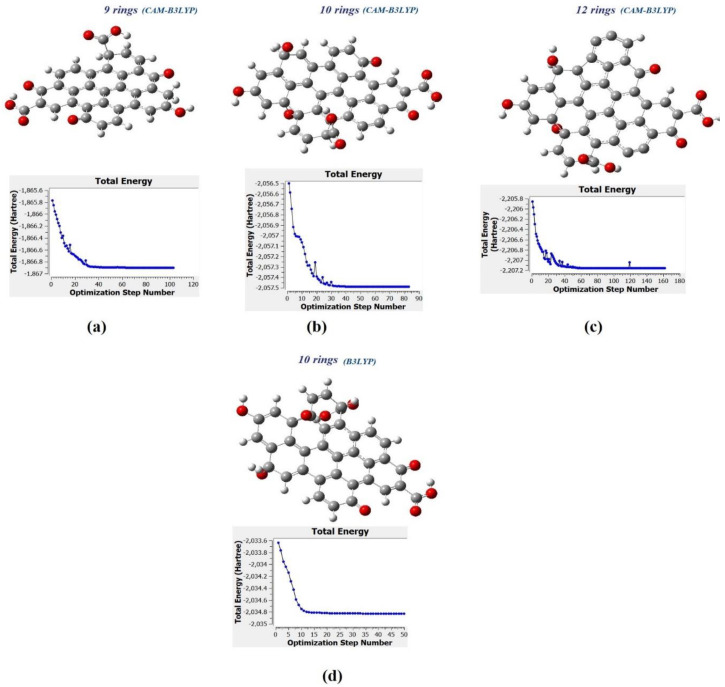
The molecular structure and energy corresponding with (**a**) CQD with 9 rings (CAM-B3LYP), (**b**) CQD with 10 rings (CAM-B3LYP), (**c**) CQD with 10 rings (B3LYP), (**d**) CQD with 12 rings (CAM-B3LYP).

**Figure 11:**
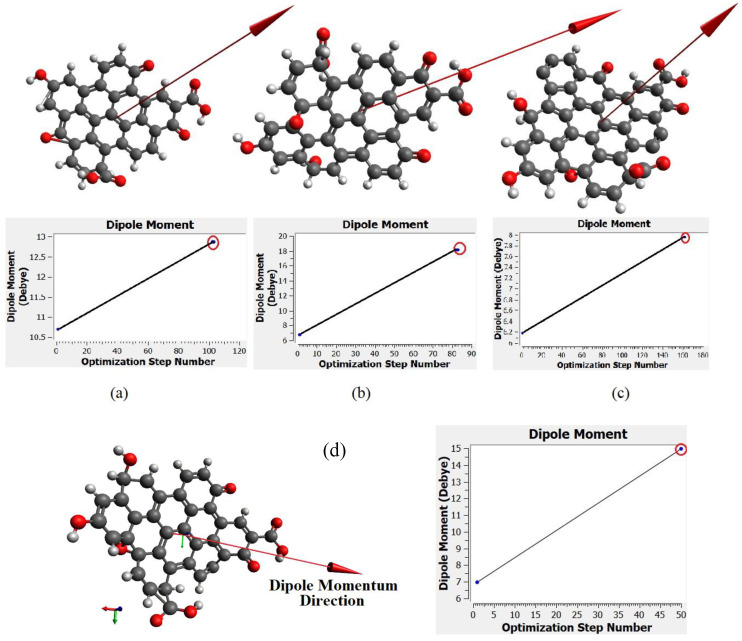
The momentum of molecule structure in the stable form related to (**a**) CQD with 9 rings (CAM-B3LYP), (**b**) CQD with 10 rings (CAM-B3LYP), (**c**) CQD with 10 rings (B3LYP), (**d**) CQD with 12 rings (CAM-B3LYP).

Figure 10a–c show the molecular structure of CQD with 9, 10, 12 rings, respectively that was obtained with the CAM-B3LYP method; and Figure 10d shows the molecule structure of CQD with 10 rings (B3LYP) that found from B3LYP method. These molecular structures are in the stable form with minimum energy equal to −1866.9022, −2057.4905, −2207.1601, and −2034.8 Hartree which were derived from Figure 10. The optimal structure was obtained when the maximum force and maximum displacement were less than threshold and the minimum energy was achieved. The pertinent parameter is summarized in Table 4. Figure 11a–d show the dipole momentum direction and amount. The dipole momentum for first and last structure can derive from each plot, and the dipole momentum were 12.8650, 18.1699, 7.9503 and 14.979 Debye, at the stable molecular structure of CQD at minimum energy. Consequently, the optimal structure was derived from DFT/CAM-B3LYP/6-31G (p).

To continue the calculation, CAM-B3YLP method was used based on 6-31G (p) for a 10-ring molecule of CQD. The optimum structure at ground state was used for Natural Bond Orbital NBO analysis. The NBO analysis is significant to investigate the intra and inter molecular bonding, interaction among bonds, and charge distribution on the surface of CQD molecule. NBO provides molecular information such as population of Lewis and non-Lewis structure, the number of core (CR), two-centre bond (BD), three-centre bond (3C), long-pair NBO, non-Lewis (NL) with high occupancy, and Lewis (L) with low-occupancy orbital as shown in Table 5. In order to obtain the acceptable Lewis structure, the occupancy of orbitals should exceed the occupancy threshold.

Hence, the analysis of CQD in the optimum structure was continued in order to obtain the natural analysis orbital (NAO) using NBO Gaussian module. The angular momentums were s, px, py, and pz and the natural orbitals type were core, valance and Rydberg [45,66,67]. The natural population of orbitals and natural charge were sorted as in the supporting data [40,49,50]. The total natural charge, natural population of core, valance and Rydberg orbitals were 0, 89.9509, 213.4373, and 0.6119, respectively. This calculation reveals that the total population of natural orbital is 304.0 and 99.8% and 0.2% of total orbital are natural minimal basis and natural Rydberg basis, respectively. The maximum occupancy is 1.99971 ≈ 2 related to core orbital (1 s) with energy about −20.2569 au and the minimum occupancy is 0.00027 and occurred in Rydberg orbital (3p) with energy equal to 1.91183. The DFT calculation depends on charge distribution on the surface of molecular. Hence, an effective valance electron configuration was achieved from the natural population of orbitals and these results are summarised in Table 6.

The occupancy of each orbital is decimal number, but the effective valance electron configuration can correspond with atomic state. For example, first carbon, first oxygen (No. 35), 39th carbon, first hydrogen (No. 44) in the Table 7 can be described by an 1 s^2^2 s^0.87^2 p^2.94^3 p^0.01^ (idealised sp^3^), 2 s^1.73^2 p^4.84^, 2 s^0.81^2 p^2.21^3 p^0.04^ (idealised sp^2^), and 1 s^0.74^ electron configuration, respectively.

## 5. Conclusions

In conclusion, we have demonstrated a facile, simple and acid-free hydrothermal extraction process in order to obtain CQD from cheap and abundant EFB biochar. The resulting hydrophilic CQD displayed a bright luminescence and excitation wavelength-dependent behavior in terms of emission intensities. The correlation between FTIR and XPS spectra shows the presence of carbonyl, carboxyl and hydroxyl functional groups in the extracted CQD, leading to the water-soluble properties of CQD. HRTEM micrographs showed the morphology of CQD to be very similar in terms of size and lattice spacing (3 nm and 0.21 nm respectively) with the EFB biochar. Based on the characterization results obtained, a hydrothermal extraction mechanism has been proposed for the formation of CQD. Furthermore, DFT calculations revealed the charge distribution between the surface functional groups and CQD where the molecular structure of CQD consists of carbonyl/carboxylate groups, which accounted for the negatively charged surface and high water-solubility of the CQD. Therefore, the excellent properties possessed by the CQD obtained via the facile hydrothermal process make it a good alternative for replacing semiconductor quantum dots, which are less favored due to their complicated synthesis process and its usage of harsh chemicals.

## Figures and Tables

**Table 1:** Comparison of carbon quantum dots (CQD) synthesis methods using hydrothermal route.

Types of Method	Carbon Sources	Process Parameter	Solvent Used	Size	Reference
Bottom-up	Banana juice	Heating - 150 °C - 4 h Centrifugation - 3000 rpm - 15 min	Ethanol	3 nm	[19]
Bottom-up	Orange juice	Heating - 120 °C - 150 min Centrifugation - 10,000 rpm - 15 min	Ethylenediamine	1.5–4.5 nm	[20]
Bottom-up	Sugarcane bagasse	Heating - 180 °C - 3 h Centrifugation - 5000 rpm - 15 min Membrane dialysis - ultrapure water - 4 days	Sodium hydroxide solution	1.8 nm	[21]
Bottom-up	Sugarcane bagasse pulp	Stirring - 24 h, room temperature Ultrasonication - 1 h Centrifugation - 10,000 rpm - 30 min	Toluene	4.1 ± 0.17 nm	[22]
Bottom-up	Soya nugget	Heating - 120 °C - 4 h	Nitric acid	5–10 nm	[23]

**Table 2:** Comparison of the quantum yield of carbon quantum dots reported in the previous studies.

Types of Carbon Quantum Dots (CQD)	Carbon Precursors from Waste	Quantum Yield (%)	References
CQD	Onion waste	28	[54]
CQD	Waste carbon paper	5.1	[55]
CQD	Lemon juice	14.86–24.89	[56]
CQD	Orange waste peels	11.37	[57]
CQD	Mango peel	8.5	[58]
CQD	EFB biochar	55.84	Present work

**Table 3:** Pertinent parameter for optimization of CQD molecular structure.

**CQD 10 Rings, 3-Parameter, Lee–Yang–Parr (B3LYP)**			
**Item**	**Value**	**Threshold**	**Converged**
Maximum Force	0.000028	0.00045	YES
root mean square RMS Force	0.000004	0.0003	YES
Maximum Displacement	0.001244	0.0018	YES
RMS Displacement	0.000212	0.0012	YES
Predicted change in Energy = −2.554014 × 10^−8^			
**CQD 9 rings, Coulomb-attenuating method-3-parameter, Lee–Yang–Parr (CAM-B3LYP)**			
**Item**	**Value**	**Threshold**	**Converged**
Maximum Force	0.000001	0.000015	YES
RMS Force	0	0.0001	YES
Maximum Displacement	0.000076	0.00006	N0
RMS Displacement	0.000011	0.00004	YES
Predicted change in Energy 3.66107 × 10^−11^			
**CQD 10 rings Coulomb-attenuating method-3-parameter, Lee–Yang–Parr (CAM-B3LYP)**			
**Item**	**Value**	**Threshold**	**Converged**
Maximum Force	0.000001	0.000015	YES
RMS Force	0	0.0001	YES
Maximum Displacement	0.000115	0.00006	N0
RMS Displacement	0.000011	0.00004	YES
Predicted change in Energy 2.5273 × 10^−11^			
**CQWD 12 rings Coulomb-attenuating method-3-parameter, Lee–Yang–Parr (CAM-B3LYP)**			
**Item**	**Value**	**Threshold**	**Converged**
Maximum Force	0	0.000015	YES
RMS Force	0	0.0001	YES
Maximum Displacement	0.000071	0.00006	N0
RMS Displacement	0.000004	0.00004	YES
Predicted change in Energy −3.7969 × 10^−12^			

**Table 4:** The minimum and maximum energy of CQD structure and dipole momentum.

Ring Number	Minimum Energy	Maximum Energy	Dipole Momentum
9	−1866.9022	−1865.7719	10.6979–12.8650
10	−2057.4905	−2056.5017	6.7069–18.1699
12	−2207.1601	−2205.8591	6.1819–7.9503

**Table 5:** Natural bond orbital of CQD with 10 rings using CAM-B3LYP methods.

Cycle	Threshold	Lewis	Non-Lewis	Number of Corecr	Two Centre Bondbd	Three-Contre Bond3c	Long Pairlp	Lewis(L)	Non-Lewis(NL)	Maximum Deviation Dev
1(1)	1.80	276.05971	19.94029	44	79	0	28	32	30	0.92
2(2)	1.80	274.05971	19.94029	44	79	0	28	32	30	0.92
3(1)	1.70	283.88905	10.11095	44	84	0	26	11	19	0.61
4(2)	1.70	283.88905	10.11095	44	85	0	26	9	19	0.61

**Table 6:** The effective valance electron based on natural population analysis.

Natural Population		
Core	89.9164	(99.9454% of 90)
Valence	216.9536	(99.7370% of 216)
Natural Minimal Basis	307.5273	(99.7987% of 307)
Natural Rydberg Basis	0.65736	(0.2013% of 307)

**Table 7:** Summary of natural population analysis.

Atom	No	Charge	Core	Valence	Rydberg	Total
C	1	0.17956	1.99621	4.70724	0.01637	6.71982
C	2	0.00952	1.99763	3.87907	0.01321	5.88991
C	3	0.16718	1.99824	3.71898	0.01696	5.73418
C	4	−0.04021	1.98900	4.23119	0.00893	6.22912
C	5	0.07513	1.99771	3.81465	0.01423	5.82659
C	6	−0.05444	1.99764	4.24075	0.01614	6.25453
C	7	0.03353	1.99753	3.85431	0.01425	5.86609
C	8	−0.02481	1.99651	4.11531	0.01531	6.12713
C	9	0.02498	1.99975	4.06476	0.01357	6.07808
C	10	−0.04376	1.99687	4.21701	0.01423	6.22811
C	11	−0.04672	1.99944	4.03583	0.01231	6.04758
C	12	−0.10680	1.99966	4.09434	0.01481	6.10881
C	13	0.58319	1.99732	3.08406	0.03400	5.11538
C	14	−0.23436	1.99788	4.32486	0.01207	6.33481
C	15	−0.03727	1.99794	4.12671	0.01282	6.13747
C	16	−0.32945	1.99698	4.31561	0.01414	6.32673
C	17	0.34903	1.99698	3.93581	0.01793	5.95072
C	18	0.03692	1.99739	3.55291	0.01246	5.56276
C	19	−0.31887	1.99975	4.61803	0.01389	6.63167
C	20	−0.12296	1.99964	4.31173	0.01374	6.32511
C	21	0.02733	1.99936	4.16133	0.01361	6.17430
C	22	−0.05951	1.99712	3.94743	0.01331	5.95786
C	23	−0.05664	1.99901	4.34814	0.01343	6.36058
C	24	−0.01151	1.99884	3.89672	0.01546	5.91102
C	25	−0.02519	1.99923	4.40411	0.01451	6.41785
C	26	−0.32224	1.99978	4.20371	0.00899	6.21248
C	27	−0.10803	1.99854	4.19983	0.01294	6.21131
C	28	−0.04752	1.99832	3.99378	0.01679	6.00889
C	29	−0.15947	1.99821	4.09283	0.01190	6.10294
C	30	−0.29763	1.99782	3.98924	0.01403	6.00109
C	31	−0.76609	1.99649	4.64875	0.00865	6.65389
C	32	−0.12459	1.99786	4.77412	0.01272	6.78470
C	33	−0.26461	1.99937	4.36552	0.01511	6.38000
C	34	0.85292	1.99897	3.17698	0.03731	5.21326
O	35	−0.57663	1.99698	6.57312	0.00176	8.57186
O	36	−0.72760	1.99878	6.17621	0.00282	8.17781
O	37	−0.67796	1.99886	6.77661	0.00452	8.77999
O	38	−0.69975	1.99969	6.98437	0.00345	8.98751
C	39	0.83923	1.99883	3.22651	0.03963	5.26497
O	40	−0.62543	1.99934	6.53173	0.00134	8.53241
O	41	−0.67961	1.99945	6.87573	0.00432	8.87950
C	42	0.54335	1.99863	3.31694	0.03487	5.35044
O	43	−0.47367	1.99933	6.56240	0.00546	8.56719
H	44	0.25369	0.00000	0.73498	0.00332	0.73830
H	45	0.34302	0.00000	0.65582	0.00116	0.65698
H	46	0.26543	0.00000	0.65248	0.00166	0.65414
H	47	0.23331	0.00000	0.80538	0.00156	0.80694
H	48	0.31240	0.00000	0.77531	0.00354	0.77885
H	49	0.30430	0.00000	0.58499	0.01101	0.59600
H	50	0.27541	0.00000	0.82461	0.00193	0.82654
H	51	0.25239	0.00000	0.64586	0.00075	0.64661
H	52	0.27052	0.00000	0.83856	0.00092	0.83948
H	53	0.42425	0.00000	0.60707	0.00535	0.61242
H	54	0.33787	0.00000	0.67843	0.00514	0.68357
H	55	0.25190	0.00000	0.75754	0.00071	0.75825
H	56	0.49684	0.00000	0.56039	0.00262	0.56301
H	57	0.45438	0.00000	0.56388	0.00220	0.56608
H	58	0.48639	0.00000	0.58883	0.00090	0.58973
H	59	0.47331	0.00000	0.62821	0.00084	0.62905
O	60	−0.54659	1.99849	6.84381	0.00302	8.84532
O	61	−0.54735	1.99899	6.73819	0.00243	8.73961
Total		0.00000	89.9164	216.9536	0.65736	307.5273
